# Simulation Study of the Effect of Influenza and Influenza Vaccination on Risk of Acquiring Guillain-Barré Syndrome

**DOI:** 10.3201/eid2102.131879

**Published:** 2015-02

**Authors:** Steven Hawken, Jeffrey C. Kwong, Shelley L. Deeks, Natasha S. Crowcroft, Allison J. McGeer, Robin Ducharme, Michael A. Campitelli, Doug Coyle, Kumanan Wilson

**Affiliations:** Institute for Clinical Evaluative Sciences, Toronto, Ontario, Canada (S. Hawken, J.C. Kwong, R. Ducharme, M.A. Campitelli, K. Wilson);; Ottawa Hospital Research Institute, Ottawa, Ontario, Canada (S. Hawken, R. Ducharme, K. Wilson);; University of Ottawa, Ottawa (S. Hawken, D. Coyle, K. Wilson);; Public Health Ontario, Toronto (J.C. Kwong, S.L. Deeks, N.S. Crowcroft);; Dalla Lana School of Public Health, University of Toronto, Toronto (J.C. Kwong, S.L. Deeks, N.S. Crowcroft, A.J. McGeer);; University of Toronto, Toronto (J.C. Kwong, N.S. Crowcroft, A.J. McGeer)

**Keywords:** seasonal influenza, influenza vaccination, Guillain-Barré syndrome, risk modeling, decision tree modeling, viruses, vaccination, influenza, modeling

## Abstract

It is unclear whether seasonal influenza vaccination results in a net increase or decrease in the risk for Guillain-Barré syndrome (GBS). To assess the effect of seasonal influenza vaccination on the absolute risk of acquiring GBS, we used simulation models and published estimates of age- and sex-specific risks for GBS, influenza incidence, and vaccine effectiveness. For a hypothetical 45-year-old woman and 75-year-old man, excess GBS risk for influenza vaccination versus no vaccination was −0.36/1 million vaccinations (95% credible interval −1.22 to 0.28) and −0.42/1 million vaccinations (95% credible interval, –3.68 to 2.44), respectively. These numbers represent a small absolute reduction in GBS risk with vaccination. Under typical conditions (e.g. influenza incidence rates >5% and vaccine effectiveness >60%), vaccination reduced GBS risk. These findings should strengthen confidence in the safety of influenza vaccine and allow health professionals to better put GBS risk in context when discussing influenza vaccination with patients.

## Abstract 2

Under typical conditions, such as influenza incidence rates of >5% and vaccine effectiveness >60%, vaccination reduced risk.

Seasonal influenza vaccination programs have been implemented in many jurisdictions over the past 40 years. Although influenza vaccination has been shown to reduce influenza-associated illness and death (*1*–*3*), there is conflicting evidence about whether influenza vaccine may increase the risk of acquiring Guillain-Barré syndrome (GBS) (*4*–*14*).

GBS is a rare but serious autoimmune condition. Most cases in European and North American populations involve acute, inflammatory, demyelinating polyneuropathy. There are several less common forms of GBS, the most frequent of which are Miller Fisher syndrome and acute motor axonal neuropathy, which are more common in Asian and Latino populations (*15*–*17*). Most GBS patients require hospitalization, ≈25% experience acute respiratory failure requiring intensive care, 10%–20% are permanently disabled, and ≈4% die within 1 year of acquiring the condition (*15*–*17*). The risk for GBS is higher in males and with increasing age: the incidence in the general population ranges from a low of 0.45/100,000 person-years in girls <10 years of age to a high of 3.7/100,000 person-years in men >80 years of age (*18*).

Many GBS cases are preceded by a respiratory or gastrointestinal infection, most commonly caused by *Campylobacter jejuni* (*16*). Recent studies have provided evidence that influenza illness is associated with the development of GBS and that influenza vaccination may confer a much more modestly increased risk of GBS than that from influenza virus infection, but these findings are less consistent (*4*–*14*). The possible association between GBS and influenza vaccination is frequently cited as a reason for vaccine refusal by health care workers (*19*–*21*). This refusal occurs despite evidence that the risk of acquiring GBS is markedly higher from influenza illness than from influenza vaccination. For example, previous studies estimate that influenza illness may increase the risk for GBS by up to 16- to 18-fold, whereas influenza vaccination may only increase the risk by up to 2-fold (*4*–*14*) (Technical Appendix Table 1).

**Table 1:** Decision tree model inputs in a simulated study of the effect of influenza and influenza vaccination on the risk of acquiring GBS*

Parameter	Expected value (95% CI)	Range of values modeled in sensitivity analyses	References†
Relative risk for GBS from influenza vaccination	1.52 (1.17–1.99)	Fixed	(*11*), online Technical Appendix Table 1
Relative risk for GBS from influenza illness	15.81 (10.28–24.32)	Fixed	(*11*), online Technical Appendix Table 1
Joint risk of influenza vaccination and influenza illness	17.33 (additive)	15.81 (subadditive) to 24.03 (multiplicative)	No available data
GBS incidence rate	0.45–3.72 cases/100,000 person years‡	0.45 in youngest girls to 3.72 in oldest men	(*23*)
Influenza illness incidence rate	10% (base case)	2%–20%	See online Technical Appendix Table 2
Vaccine effectiveness		20%–80%	(*3**, **25**–**27*)
<65 y of age	0.61 (0.30–0.52)		
>65 y of age	0.50 (0.27–0.91)		

*GBS, Guillain-Barré syndrome. †The online Technical Appendix is available at http://wwwnc.cdc.gov/EID/article/2/21/13-1879-Techapp1.pdf. ‡Depending on age and sex.

On balance, it is unclear whether vaccination against seasonal influenza results in a net increase or decrease in the absolute risk of a person acquiring GBS. If influenza vaccination results in a small increased risk while reducing the incidence of influenza illness (which confers a much larger increase in GBS risk), then the net effect of vaccination could be a reduction in the absolute risk of GBS. The objective of this study was to assess, by using a simulation modeling approach, the effect of receipt of a seasonal influenza vaccine on a person’s age- and sex-specific absolute risk of acquiring GBS.

## Methods

### Intervention and Study Design

**Figure 1:**
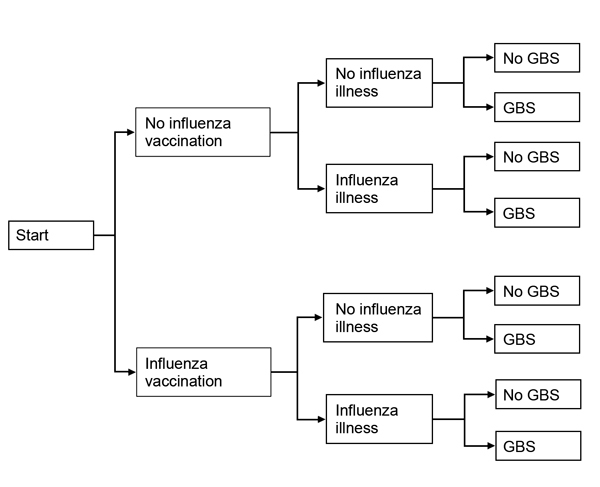
Probabilistic decision tree modeling approach used in a study simulating the effect of influenza and influenza vaccination on the risk of acquiring Guillain-Barré syndrome (GBS). It is assumed that each person has the choice of being vaccinated against influenza.

We compared the net impact of receiving a seasonal influenza vaccine versus not receiving the vaccine on a person’s risk of acquiring GBS. We used a probabilistic decision tree modeling approach, in which each person was faced with the choice of receiving a vaccination against influenza (Figure 1). Using effect estimates and associated standard errors and/or confidence intervals from recent peer-reviewed literature, we simulated observations and modeled uncertainty by using appropriate distributional assumptions based on the type of effect estimate (e.g., relative risk [RR], incidence rate). We defined 2 base-case examples (a 45-year-old woman and a 75-year-old man) and then performed a series of sensitivity analyses to demonstrate the effect of important covariates on the risk for acquiring GBS.

### Model Inputs

Age- and sex-specific baseline risks for GBS were based on a published regression model derived from a meta-analysis of studies reporting GBS incidence (*18*). We calculated age- and sex-specific individual GBS risk estimates for influenza incidence rates ranging from 2% to 20% and for estimates of vaccine effectiveness ranging from 20% to 80%. Published RR estimates for GBS with respect to influenza vaccination and influenza-like illness are listed in Technical Appendix Table 1. We used the GBS risk estimates reported in Kwong et al. (*11*) in our simulation models; these estimates were consistent with findings of other studies (Technical Appendix Table 1).

Given that influenza illness and vaccination are only 2 (relatively minor) transient contributors to the overall risk for GBS, we assumed that the risk increase for GBS persisted for only 6 weeks following exposure to both influenza illness and vaccination (*4*,*5*,*9*–*13*). A person’s risk for seasonal influenza illness depends on age, vaccination status, geographic location, and sociodemographic factors. Estimates of influenza incidence also vary widely by year and case definition (i.e., confirmed by culture, PCR, or serologic testing). Published estimates of laboratory-confirmed influenza incidence in unvaccinated persons are listed in Technical Appendix Table 2. Because of the observed annual variability in influenza incidence, we modeled a wide range (2%–20%) of rates in sensitivity analyses. Similarly, given that vaccine effectiveness varies by recipient age, type of vaccine (e.g., trivalent inactivated vaccine vs. live attenuated influenza vaccine), and success of matching the vaccine strains to circulating strains, we considered a range (20%–80%) of effectiveness estimates.

**Table 2:** Excess risk for GBS per million influenza vaccinations overall and for males and females separately by various influenza incidence rates in a simulated study*

Age, y, sex	∆GBS risk (95% CrI), % ΔGBS risk <0, by influenza incidence rate†				
	2%	5%	10%	15%	20%
45					
Both	0.49 (−0.03 to 1.35), 3.5^(+)^	0.12 (−0.55 to 0.93), 35.7^(±)^	−0.48 (−1.63 to 0.37), 87.1^(−)^	−1.08 (−2.79 to −0.07), 98.2^(−)^	−1.69 (−3.99 to −0.43), 99.7^(−)^
F	0.37 (−0.02 to 1.01), 3.5^(+)^	0.09 (−0.41 to 0.70), 35.6^(±)^	−0.36 (−1.22 to 0.28),‡ 87.0^(−)^	−0.82 (−2.09 to −0.05), 98.2^(−)^	−1.28 (−2.98 to −0.33), 99.7^(−)^
M	0.66 (−0.05 to 1.79), 3.5^(+)^	0.16 (−0.74 to 1.24), 35.6^(±)^	−0.65 (−2.16 to 0.50), 87.1^(−)^	−1.46 (−3.69 to −0.09), 98.1^(−)^	−2.28 (−5.27 to −0.59), 99.7^(−)^
75					
Both	0.90 (0.19 to 2.71), 2.2^(+)^	0.43 (−0.79 to 2.20), 22.6^(+)^	−0.31 (−2.58 to 1.74), 64.7^(±)^	−1.07 (−4.55 to 1.51), 82.6^(−)^	−1.84 (−6.60 to 1.38), 89.3^(−)^
F	0.69 (0.02 to 2.18), 2.3^(+)^	0.32 (−0.61 to 1.77), 22.6^(+)^	−0.23 (−2.06 to 1.37), 64.7^(±)^	−0.80 (−3.67 to 1.16), 82.5^(−)^	−1.39 (−5.33 to 1.05), 89.3^(−)^
M	1.23 (0.02 to 3.90), 2.3^(+)^	0.58 (−1.09 to 3.15), 22.6^(+)^	−0.42 (−3.68 to 2.44),‡ 64.8^(±)^	−1.44 (−6.54 to 2.09), 82.6^(−)^	−2.48 (−9.47 to 1.89), 89.2^(−)^

*Assuming vaccine effectiveness of 61% for 45-year-old persons and 50% for 75-year-old persons. Assuming semi-additive effect whereby those vaccinated and who experience influenza illness experience the sum of the 2 relative risks (RR) (i.e., influenza illness RR = 15.81 + 1.52 = 17.33). A total of 1,000,000 simulations were conducted for each scenario. GBS, Guillain-Barré syndrome. Explanations for superscript symbols: (+) <25% of estimates have ∆GBS <0 (favors vaccination increasing GBS risk); (±) 25%–75% of estimates have ∆GBS <0 (neutral); (−) >75% of estimates have ∆GBS <0 (favors vaccination decreasing GBS risk). †Absolute risk difference between vaccinated and unvaccinated persons. Negative values for ∆ GBS indicate net reduction in no. of GBS cases in vaccinated vs. unvaccinated persons. The % of ∆GBS <0 is the percentage of simulation results where the absolute risk difference for vaccinated vs. unvaccinated was <0 (i.e., protective). ‡Base-case analyses.

### Joint Effects of Influenza Vaccination and Illness

Because influenza vaccination may fail to prevent influenza illness, we considered 3 possible scenarios to model the joint effects of vaccination and influenza illness. If exposure to both influenza vaccination and illness occurred, it would be possible for the two 6-week risk periods to overlap. To simplify our simulation, we assumed a single 6-week exposure period for the 2 exposures combined. We varied the joint RR of influenza illness and vaccination to assess conditions of overlapping risk periods and interaction between exposures.

In the first scenario considered, we modeled exposures as independent and non-overlapping. Thus, the absolute risk of GBS would be equivalent to an additive joint effect on the RR scale: joint GBS risk = RR_(influenza vaccination)_ × (6-wk baseline GBS incidence rate) + RR_(influenza illness)_ × (6-wk baseline GBS incidence rate) = (RR_(influenza vaccination)_ + RR_(influenza illness)_) × (6 week baseline GBS incidence rate). We chose this additive joint risk model for our base-case simulations and then used different joint risk models in sensitivity analyses.

In the second scenario, we assumed the joint risk to be no higher than the risk of influenza illness alone: joint GBS risk = RR_(influenza illness)_ × (6-wk baseline GBS incidence rate). In the third scenario, we assumed that the joint risk was multiplicative on the RR scale: joint GBS risk = (RR_(influenza vaccination)_) × (RR_(influenza illness)_) × (6-wk baseline GBS incidence rate).

### Modeling Approach

Technical details of our methodologic approach for generating representative simulated observations are provided in the Technical Appendix. All simulation parameters and associated statistical uncertainty (i.e., standard errors) were chosen to reflect current peer-reviewed evidence. We simulated 1,000,000 observations each for vaccinated and unvaccinated persons and calculated the absolute risk difference with respect to GBS for the 2 scenarios. We then calculated the median and a 95% credible interval (CrI), defined as the region between percentiles 2.5 and 97.5 of the 1,000,000 simulated risk differences for each scenario. Point estimates for each of the model inputs were used to calculate absolute risk differences for deterministic sensitivity analyses.

### Base-Case Analyses

We conducted 2 base-case analyses to represent the situation of typical persons faced with the decision of whether to receive the influenza vaccine. We first modeled the risk of GBS for a 45-year-old woman with a baseline risk for GBS of 0.97/100,000 person-years (95% CI 0.62–1.53) (*18*), a 10% chance of influenza illness (if unvaccinated), and vaccine effectiveness (if vaccinated) of 61% (95% CI 30%–52%) for a hypothetical trivalent inactivated vaccine (*3*,*22*). We then conducted a similar analysis for a 75-year-old man with a baseline risk for GBS of 3.07/100,000 person-years (95% CI 1.50–6.27) (*18*), a 10% chance of influenza illness (if unvaccinated), and a vaccine effectiveness (if vaccinated) of 50% (95% CI 27%–91%) for the same hypothetical vaccine (*23*).

### Sensitivity Analyses

In sensitivity analyses, model inputs (influenza incidence rate, joint effect of vaccination and influenza illness on GBS risk, vaccine effectiveness, and sex) were changed one at a time to determine the effect of higher and lower plausible values on excess risk for GBS with vaccination. We constructed tornado plots to display the effect of each factor we changed (*24*). The tornado plot displays the results of 1-way sensitivity analyses, which illustrate the effect of high and low values for each variable of interest while fixing all other variables at their respective base-case point estimates in the influenza GBS risk model. The tornado plots are displayed as stacked bar charts, with covariates ranked from most impactful at the top and the least impactful at the bottom, thus giving them the appearance of a tornado funnel. We then performed another series of sensitivity analyses involving 3-way sensitivity plots of excess risk for GBS by age (<18, 45, 60, and 75 years), vaccine effectiveness, and incidence of influenza illness, averaged over both sexes.

All expected values, measures of uncertainty for each, and ranges of inputs that we used in the base-case and sensitivity analyses are reported in Table 1. All simulations and statistical graphics were conducted in R version 3.0.1 (http://www.r-project.org/).

## Results

### Base-Case Analyses

When a 45-year-old woman was used as the base case, excess GBS risk for influenza vaccination versus no vaccination was calculated to be −0.36/1 million vaccinations (95% CrI −1.22% to 0.28%), representing a small absolute reduction in GBS risk. Most (87%) of the simulated absolute risk differences were <0, indicating that vaccination is (slightly) protective against GBS. When a 75-year-old man was used as the base case, the excess GBS risk due to vaccination was estimated to be –0.42/1 million vaccinations (95% CrI –3.68 to 2.44), and 65% of simulated absolute risk differences were <0. Absolute risk differences are presented in Table 2 for influenza incidence rates ranging from 2% to 20%.

### Sensitivity Analyses

**Figure 2:**
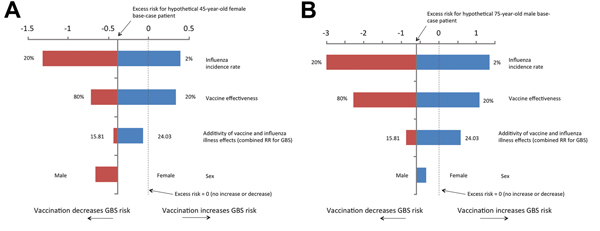
Sensitivity analyses for the excess risk of Guillain-Barré syndrome (GBS) per 1,000,000 influenza vaccinations. A) 45-year-old woman, assuming a 10% influenza incidence rate, 61% vaccine effectiveness, and combined relative risk (RR) of GBS of 17.33. B) 75-year-old man, assuming a 10% influenza incidence rate, vaccine effectiveness of 50% and combined RR of GBS of 17.33. Depending on the joint distribution of the probabilistic inputs to the simulation, these deterministic sensitivity analyses will not necessarily yield identical mean/median estimates to those from the probabilistic simulation for the same age, sex, and influenza incidence rate.

In Figure 2, we present tornado plots that illustrate the relative influence of varying each model input (influenza incidence, vaccine effectiveness, sex, and joint RRs for vaccination and influenza illness) on the excess risk of GBS while holding all other factors fixed. For both base cases, influenza incidence and vaccine effectiveness were the most influential factors. Under most typical conditions, vaccination was protective against GBS; under conditions for which the model predicted an increased absolute risk, the excess GBS risk did not exceed approximately 1 in 1 million (Figure 2). The only exceptions noted were for the 75-year-old male base case when influenza incidence approached a low of 2% and when vaccine effectiveness was only 20%. For 45- and 75-year-old men and women (averaged over sex), when the influenza incidence rate was held constant at 10%, the threshold for protection from GBS with vaccination was crossed when the vaccine was at least 39% effective. Conversely, if vaccine effectiveness was held fixed at 61% in 45-year-old men and women and at 50% in 75-year-old men and women (*26*,*27*), the threshold for protection with vaccination was crossed when the influenza incidence rate was at least 6% and 7.5%, respectively. When the joint effect of influenza vaccination and illness were modeled as multiplicative on the RR scale, the benefit of vaccination on individual risk of GBS was muted or absent, compared with the benefit in the more plausible scenarios, in which 1) exposures were either independent and non-overlapping (additive joint effect on relative risk scale) or 2) joint exposure conferred the same risk as exposure to influenza illness alone (Figure 2).

**Figure 3:**
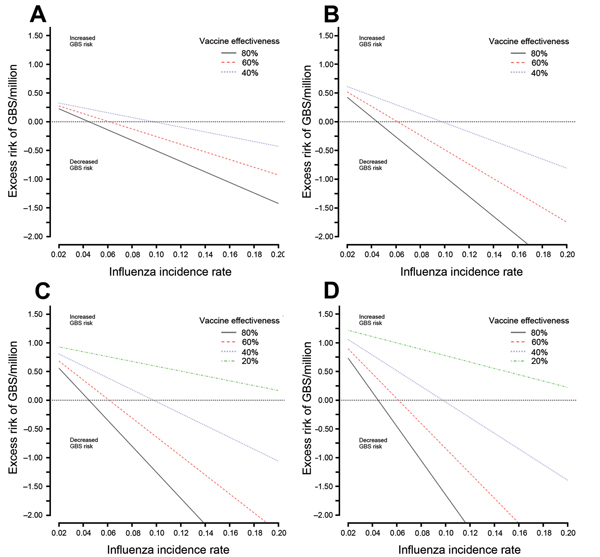
Excess risk of Guillain-Barré syndrome (GBS) per 1,000,000 influenza vaccinations by influenza incidence rate, age, and vaccine effectiveness for both sexes combined. A) Risk for persons <18 years of age; vaccine effectiveness of 40%–80%. B) Risk for persons 45 years of age; vaccine effectiveness of 40%–80%. C) Risk for persons 60 years of age; vaccine effectiveness of 20%–80%. D) Risk for persons 75 years of age; vaccine effectiveness of 20%–80%. Depending on the joint distribution of the probabilistic inputs to the simulation model, these deterministic sensitivity analyses will not necessarily yield identical mean/median estimates to those from the probabilistic simulation for the same age, sex, and influenza incidence rate.

In Figure 3, we separately present 3-way sensitivity analyses of absolute GBS risk by influenza incidence rate and vaccine effectiveness for each age group. When vaccine effectiveness was 60%, vaccination was protective in all age groups for influenza incidence rates of ≈6% or higher. Overall, the observed net benefit of vaccination on the risk for GBS was strongest with high vaccine effectiveness in older persons (for modeled scenarios in which vaccine effectiveness remained similar to that for younger persons) and among males because of their higher baseline incidence of GBS (not shown). For the lower limit of vaccine effectiveness of 20% considered for elderly subjects (ages 60 and 75, Figure 3, panels C, D), the excess risk of GBS was positive (favoring no vaccination) even for influenza incidence values as high as 20% (the highest incidence considered).

An online tool has been developed to allow readers to calculate individual GBS risk for a range of model inputs. The tool implements the deterministic model used to calculate the results presented in Figures 2 and 3 (http://www.stevenhawken.ca/Software_and_Web_Tools.html).

## Discussion

To better understand the complex relationship between influenza vaccination and influenza illness with respect to GBS risk, we constructed probabilistic decision tree simulation models to evaluate the risk of GBS for a person who either does or does not receive seasonal influenza vaccine. Our simulations provide evidence that, under many conditions, vaccination is more likely to reduce rather than increase a person’s overall risk of acquiring GBS. The most important factors in determining the net benefit or harm were the influenza incidence rate and vaccine effectiveness. Hence, when low influenza incidence was coupled with poor vaccine effectiveness, our models predicted a net increased risk of GBS with vaccination. Low influenza incidence and low vaccine effectiveness are not necessarily uncommon. For example, vaccine effectiveness tends to be lower in elderly persons, and incidence rates fluctuate from year to year. Where vaccine coverage and, by extension, herd immunity is high, influenza incidence rates will be lower. Even when both vaccine effectiveness and influenza incidence rates were low, the absolute risk increases observed in our simulations under these conditions were extremely small. In all but the most extreme cases, the excess risk with vaccination did not exceed the generally quoted figure of 1 in 1 million (*5*).

Although our investigation focused on individual risk, our results can potentially be interpreted from a population health perspective. For example, an excess risk of 1 in 1 million can be interpreted as the number of excess cases of GBS expected for every 1,000,000 people choosing to be vaccinated under the stated assumptions. The effect of incomplete vaccine uptake and herd immunity would serve to lower the effective number of persons vaccinated and lower the effective influenza incidence, which could be addressed in sensitivity analyses rather than by explicitly modeling them. These phenomena would have greatly complicated a simulation study focused at the population health level, but such complications were avoided in our study by focusing on individual persons.

Although local estimates of influenza incidence vary widely by year, age, geographic location, and sociodemographic factors, the World Health Organization estimates that overall, 20%–30% of children and 5%–10% of adults are affected by influenza illness annually (*28*). A recent meta-analysis reported seasonal influenza incidence rates of 5.4% (95% CI 3.0%–9.8%) in unvaccinated working adults, 24.2% (95% CI 15.1%–38.9%) in unvaccinated working adults living in households with children, and 18.7% (95% CI 15.8%–22.1%) in unvaccinated health care workers (*29*). This wide range of reported influenza incidence rates motivated our decision to use various influenza incidence rates to encompass the spectrum of plausible values.

Data from observational studies suggest that influenza vaccines may be less effective in elderly persons (*23*). This reduced effectiveness could result in a muted benefit of vaccination with respect to GBS risk, a fact that we confirmed in our simulations. However, higher baseline rates of GBS are observed in the elderly, so a vaccine with reduced efficacy could still be protective with respect to GBS risk. Furthermore, if vaccine effectiveness is reduced because of lowered immunogenicity (as distinct from poor antigen match), the risk for GBS from vaccination could be lowered if GBS risk is correlated with the immune response mounted by the vaccinee.

Vaccine effectiveness estimates are also heterogeneous by year and region and are dependent on antigen match between the vaccine and circulating virus strains. We found that when the annual influenza incidence was held fixed at 10%, vaccination was protective against GBS when vaccine effectiveness was at least 39%. Although Canadian annual adjusted vaccine effectiveness estimates from 2005–06 to 2010–11 have ranged from 37% (95% CI 17%–52%) to 61% (95% CI 26%–79%), estimates were >45% for every season except 2010–11 (*25*,*30*–*32*). A recent study from the United States reported a vaccine effectiveness of 60% (95% CI 53%–66%) for the 2010–11 season; the estimate for children 6 months to 8 years of age was 69%, and that for adults >65 years of age was 38% (*33*). For the 2011–12 season, a study from the United Kingdom reported a vaccine effectiveness of 23% (95% CI−10% to 47%) (*26*). For the same season, a European study reported estimates of 63% (95% CI 26%–82%) for persons 15–59 years of age and markedly lower estimates for younger (19%) and older (15%) persons (*34*). Despite their heterogeneity, these estimates of influenza incidence rates and vaccine effectiveness support our conclusion that under typical conditions, vaccination against seasonal influenza will result in a net decrease in absolute risk for GBS.

Previous studies of the risk for GBS from seasonal influenza vaccination and illness either looked solely at vaccination (*4*–*6*) or considered seasonal influenza illness and vaccination separately within the same study (*7*,*8*,*11*). Several studies looked at the risk for GBS from influenza A(H1N1) virus infection and vaccination (*27*,*35*,*36*). Our study considered the effect of seasonal influenza vaccination and influenza illness on GBS risk simultaneously, while taking into account the effect of vaccine effectiveness on reducing the incidence of influenza illness, as well as the important roles of age and sex on baseline risk for GBS, influenza incidence, and vaccine effectiveness.

This study has important strengths and limitations. A strength of our study is that we were able to model excess risk of GBS for a wide range of scenarios. We were also able to account for different assumptions about the combined effects of influenza illness and vaccination in modeling the joint risk of GBS if influenza illness were to occur in persons who had been vaccinated. We based all model inputs on recent peer-reviewed evidence.

One potential limitation of our study is that the principal studies quantifying the risk of GBS with influenza illness ascertained cases of influenza in different ways. Many of the studies reporting on vaccine safety and efficacy used medically attended, laboratory-confirmed influenza illness as the primary outcome (*3*,*22*,*23*,*37*). Estimates of GBS risk have been almost exclusively derived from studies of association with influenza-like illness; one exception is an ecologic study that did not provide an estimate of RR (*38*). It is unclear whether the risk for GBS (and influenza vaccine effectiveness) is similar for laboratory-confirmed influenza and influenza-like illness or influenza-coded health care encounters. Although the incidence of laboratory-confirmed influenza underestimates the true burden of influenza illness, influenza-coded outpatient visits are vulnerable to misclassification.

The excess risk calculated in our model is sensitive to the estimated combined effect of influenza illness and vaccination on the RR for GBS. At one extreme, we assumed that the effects were multiplicative on the RR scale, and at the other extreme, we assumed the combined risk was no higher than the RR for influenza illness alone. It is possible that GBS risk varies by severity of illness, such that asymptomatic or minimally symptomatic illness confers a lower risk of GBS than more severe influenza-like illness; if this is the case, we may have overestimated influenza-associated GBS risk. It is also plausible that vaccination could reduce the severity of subsequent influenza illnesses that are not altogether prevented, and in turn, could reduce the risk of GBS due to those illnesses. Similarly, when vaccine effectiveness is reduced because of immunogenicity and not poor antigen match, the risk of GBS from vaccination could actually decrease if the risk is linked to the immune response mounted by the vaccinee. In our search of the literature, we did not find data on the severity of illness from influenza or other pathogens in relation to risk for GBS or on the combined risk of influenza vaccination and influenza illness on risk for GBS. Future studies exploring these questions would be welcome, although achieving the necessary statistical power would be extremely challenging.

In conclusion, our findings provide reassurance that influenza vaccination reduces individual risk of GBS except under conditions of low influenza incidence and/or low vaccine effectiveness. Even under those circumstances, in which the absolute risk of GBS may be raised by vaccination, the excess risk is small (in most cases, less than the generally quoted estimate of 1 in 1 million). The protective benefits of influenza vaccination are most pronounced for populations in which influenza incidence rates are higher (i.e., young children and the elderly, although effectiveness may be muted) and in those with a higher background risk for GBS (males and older persons). Influenza vaccination is an important population health intervention that reduces morbidity and mortality. Beyond these benefits, the tendency of influenza vaccination to reduce a person’s overall risk of acquiring GBS under many conditions (although the absolute risk differences are extremely small) should strengthen confidence in the safety of influenza vaccination and allow health professionals to better put the risk of GBS in context when communicating risks and benefits to potential vaccinees.

Technical Appendix Details of the simulation modeling approach and tables showing estimated risks from other studies and influenza incidence rates from observational studies and randomized controlled trials.

## References

[1] Kwong JC, Stukel TA, Lim J, McGeer AJ, Upshur REG, Johansen H, The effect of universal influenza immunization on mortality and health care use. PLoS Med. 2008;5:e211. 10.1371/journal.pmed.0050211 18959473 PMC2573914

[2] Manzoli L, Ioannidis JPA, Flacco ME, De Vito C, Villari P. Effectiveness and harms of seasonal and pandemic influenza vaccines in children, adults and elderly: a critical review and re-analysis of 15 meta-analyses. Hum Vaccin Immunother. 2012;8:851–62. 10.4161/hv.19917 22777099 PMC3495721

[3] Osterholm MT, Kelley NS, Sommer A, Belongia EA. Efficacy and effectiveness of influenza vaccines: a systematic review and meta-analysis. Lancet Infect Dis. 2012;12:36–44. 10.1016/S1473-3099(11)70295-X 22032844

[4] Lasky T, Terracciano GJ, Magder L, Koski CL, Ballesteros M, Nash D, The Guillain-Barré syndrome and the 1992–1993 and 1993–1994 influenza vaccines. N Engl J Med. 1998;339:1797–802. 10.1056/NEJM199812173392501 9854114

[5] Juurlink DN, Stukel TA, Kwong J, Kopp A, McGeer A, Upshur RE, Guillain-Barré syndrome after influenza vaccination in adults: a population-based study. Arch Intern Med. 2006;166:2217–21. 10.1001/archinte.166.20.2217 17101939

[6] Hughes RA, Charlton J, Latinovic R, Gulliford MC. No association between immunization and Guillain-Barré syndrome in the United Kingdom, 1992 to 2000. Arch Intern Med. 2006;166:1301–4. 10.1001/archinte.166.12.1301 16801513

[7] Tam CC, O'Brien SJ, Petersen I, Islam A, Hayward A, Rodrigues LC. Guillain-Barré syndrome and preceding infection with campylobacter, influenza and Epstein-Barr virus in the general practice research database. PLoS ONE. 2007;2:e344. 10.1371/journal.pone.0000344 17406668 PMC1828628

[8] Stowe J, Andrews N, Wise L, Miller E. Investigation of the temporal association of Guillain- Barré syndrome with influenza vaccine and influenzalike illness using the United Kingdom General Practice Research Database. Am J Epidemiol. 2009;169:382–8. 10.1093/aje/kwn310 19033158

[9] Greene SK, Rett M, Weintraub ES, Li L, Yin R, Amato AA, Risk of confirmed Guillain- Barré syndrome following receipt of monovalent inactivated influenza A (H1N1) and seasonal influenza vaccines in the Vaccine Safety Datalink Project, 2009–2010. Am J Epidemiol. 2012;175:1100–9. 10.1093/aje/kws195 22582210 PMC6272801

[10] Wise ME, Viray M, Sejvar JJ, Lewis P, Baughman AL, Connor W, Guillain- Barré syndrome during the 2009–2010 H1N1 influenza vaccination campaign: population-based surveillance among 45 million Americans. Am J Epidemiol. 2012;175:1110–9. 10.1093/aje/kws196 22582209 PMC3888111

[11] Kwong JC, Vasa P, Campitelli MA, Hawken S, Wilson K, Rosella LC, Risk of Guillain-Barré syndrome after seasonal influenza vaccination and influenza health-care encounters: a self-controlled study. Lancet Infect Dis. 2013;13:769–76. 10.1016/S1473-3099(13)70104-X 23810252

[12] Galeotti F, Massari M, D’alessandro R, Beghi E, Chiò A, Logroscino G, Risk of Guillain-Barré syndrome after 2010–2011 influenza vaccination. Eur J Epidemiol. 2013;28:433–44. 10.1007/s10654-013-9797-8 23543123 PMC3672511

[13] Baxter R, Bakshi N, Fireman B, Lewis E, Ray P, Vellozzi C, Lack of association of Guillain- Barré syndrome with vaccinations. Clin Infect Dis. 2013;57:197–204. 10.1093/cid/cit222 23580737

[14] Vellozzi C, Iqbal S, Broder K. Guillain-Barré syndrome, influenza, and influenza vaccination: the epidemiologic evidence. Clin Infect Dis. 2014;58:1149–55. 10.1093/cid/ciu005 24415636

[15] Hughes RAC, Cornblath DR. Guillain-Barré syndrome. Lancet. 2005;366:1653–66. 10.1016/S0140-6736(05)67665-9 16271648

[16] Yuki N, Hartung H-P. Guillain-Barré syndrome. N Engl J Med. 2012;366:2294–304. 10.1056/NEJMra1114525 22694000

[17] Hughes RA, Rees JH. Clinical and epidemiologic features of Guillain-Barré syndrome. J Infect Dis. 1997;176(Suppl 2):S92–8. 10.1086/513793 9396689

[18] Sejvar JJ, Baughman AL, Wise M, Morgan OW. Population incidence of Guillain-Barré syndrome: a systematic review and meta-analysis. Neuroepidemiology. 2011;36:123–33. 10.1159/000324710 21422765 PMC5703046

[19] Zhang J, While AE, Norman IJ. Nurses’ knowledge and risk perception towards seasonal influenza and vaccination and their vaccination behaviours: a cross-sectional survey. Int J Nurs Stud. 2011;48:1281–9. 10.1016/j.ijnurstu.2011.03.002 21474136

[20] Chor JSY, Pada SK, Stephenson I, Goggins WB, Tambyah PA, Clarke TW, Seasonal influenza vaccination predicts pandemic H1N1 vaccination uptake among healthcare workers in three countries. Vaccine. 2011;29:7364–9. 10.1016/j.vaccine.2011.07.079 21807048

[21] Prematunge C, Corace K, McCarthy A, Nair RC, Pugsley R, Garber G. Factors influencing pandemic influenza vaccination of healthcare workers—a systematic review. Vaccine. 2012;30:4733–43. 10.1016/j.vaccine.2012.05.018 22643216

[22] Jefferson T, Di Pietrantonj C, Rivetti A, Bawazeer GA, Al-Ansary LA, Ferroni E. Vaccines for preventing influenza in healthy adults. Cochrane Database Syst Rev. 2010;CD001269 . 20614424 10.1002/14651858.CD001269.pub4

[23] Jefferson T, Di Pietrantonj C, Al-Ansary LA, Ferroni E, Thorning S, Thomas RE. Vaccines for preventing influenza in the elderly. Cochrane Database Syst Rev. 2010;CD004876 . 20166072 10.1002/14651858.CD004876.pub3

[24] Eschenbach T. Technical note: constructing tornado diagrams with spreadsheets. Eng Economist. 2006;51:195–204. 10.1080/00137910600695676

[25] Janjua NZ, Skowronski DM, De Serres G, Dickinson J, Crowcroft NS, Taylor M, Estimates of influenza vaccine effectiveness for 2007–2008 from Canada's sentinel surveillance system: cross-protection against major and minor variants. J Infect Dis. 2012;205:1858–68. 10.1093/infdis/jis283 22492921

[26] Pebody R, Andrews N, McMenamin J, Durnall H, Ellis J, Thompson CI, Vaccine effectiveness of 2011/12 trivalent seasonal influenza vaccine in preventing laboratory-confirmed influenza in primary care in the United Kingdom: evidence of waning intra-seasonal protection. Euro Surveill. 2013;18:20389 . 23399424 10.2807/ese.18.05.20389-en

[27] Greene SK, Rett MD, Vellozzi C, Li L, Kulldorff M, Marcy SM, Guillain-Barré syndrome, influenza vaccination, and antecedent respiratory and gastrointestinal infections: a case-centered analysis in the Vaccine Safety Datalink, 2009–2011. PLoS ONE. 2013;8:e67185. 10.1371/journal.pone.0067185 23840621 PMC3694016

[28] World Health Organization. International travel and health: seasonal influenza [cited 2013 Sep 9]. http://www.who.int/ith/diseases/influenza_seasonal/en

[29] Kuster SP, Shah PS, Coleman BL, Lam P-P, Tong A, Wormsbecker A, Incidence of influenza in healthy adults and healthcare workers: a systematic review and meta-analysis. PLoS ONE. 2011;6:e26239. 10.1371/journal.pone.0026239 22028840 PMC3196543

[30] Skowronski DM, De Serres G, Dickinson J, Petric M, Mak A, Fonseca K, Component-specific effectiveness of trivalent influenza vaccine as monitored through a sentinel surveillance network in Canada, 2006–2007. J Infect Dis. 2009;199:168–79. 10.1086/595862 19086914

[31] Skowronski DM, Janjua NZ, De Serres G, Winter AL, Dickinson JA, Gardy JL, A Sentinel platform to evaluate influenza vaccine effectiveness and new variant circulation, Canada 2010–2011 season. Clin Infect Dis. 2012;55:332–42 and. 10.1093/cid/cis431 22539661

[32] Skowronski DM, De Serres G, Crowcroft NS, Janjua NZ, Boulianne N, Hottes TS, Association between the 2008–09 seasonal influenza vaccine and pandemic H1N1 illness during spring–summer 2009: four observational studies from Canada. PLoS Med. 2010;7:e1000258. 10.1371/journal.pmed.1000258 20386731 PMC2850386

[33] Treanor JJ, Talbot HK, Ohmit SE, Coleman LA, Thompson MG, Cheng P-Y, Effectiveness of seasonal influenza vaccines in the United States during a season with circulation of all three vaccine strains. Clin Infect Dis. 2012;55:951–9. 10.1093/cid/cis574 22843783 PMC3657521

[34] Kissling E, Valenciano MM, Larrauri AA, Oroszi BB, Cohen JJ, Nunes BB, Low and decreasing vaccine effectiveness against influenza A(H3) in 2011/12 among vaccination target groups in Europe: results from the I-MOVE multicentre case-control study. Euro Surveill. 2013;18:20390 . 23399425 10.2807/ese.18.05.20390-en

[35] Grimaldi-Bensouda L, Alpérovitch A, Besson G, Vial C, Cuisset J-M, Papeix C, Guillain- Barré syndrome, influenzalike illnesses, and influenza vaccination during seasons with and without circulating A/H1N1 viruses. Am J Epidemiol. 2011;174:326–35. 10.1093/aje/kwr072 21652600

[36] Dieleman J, Romio S, Johansen K, Weibel D, Bonhoeffer J, Sturkenboom M, Guillain- Barré syndrome and adjuvanted pandemic influenza A (H1N1) 2009 vaccine: multinational case-control study in Europe. BMJ. 2011;343:d3908. 10.1136/bmj.d3908 21750072 PMC3134565

[37] Jefferson T, Rivetti A, Di Pietrantonj C, Demicheli V, Ferroni E. Vaccines for preventing influenza in healthy children. Cochrane Database Syst Rev. 2012;8:CD004879 . 22895945 10.1002/14651858.CD004879.pub4 PMC6478137

[38] Tam CC, O'Brien SJ, Rodrigues LC. Influenza, *Campylobacter* and *Mycoplasma* infections, and hospital admissions for Guillain-Barré syndrome, England. Emerg Infect Dis. 2006;12:1880–7 . 10.3201/eid1212.051032 17326939 PMC3291336

