# Supplementary material for: Simulation Study of the Effect of Influenza and Influenza Vaccination on Risk of Acquiring Guillain-Barré Syndrome
Source: Emerg Infect Dis. 2015 Feb;21(2):224–31. doi: 10.3201/eid2102.131879 (PMC4313628; doi:10.3201/eid2102.131879)
Supplement: Technical Appendix — Details of the simulation modeling approach and tables showing estimated risks from other studies and influenza incidence rates from observational studies and randomized controlled trials. [file 13-1879-Techapp-s1.pdf]

Article DOI: http://dx.doi.org/10.3201/eid2102.131879

Simulation Study of the Effect of Influenza and Influenza Vaccination on Risk of Acquiring Guillain-Barré Syndrome

Technical Appendix

Details of Simulation Modeling Approach

To simulate observations probabilistically, based on a published relative risk point estimates and 95% CIs, a lognormal distribution was assumed. The mean and standard error on the log scale were then back-calculated from the point estimate and 95% CI and then random draws were taken from the normal distribution with the corresponding mean and standard deviation. These random draws were then exponentiated to yield observations on the original relative risk scale (*1*,*2*). For rates, we took advantage of the fact that the β distribution is a conjugate to binomial data, such that we can draw simulated observations based on a rate and its standard error from a β distribution with appropriately derived shape parameters (*1*,*2*). All simulation parameters and associated statistical uncertainty (i.e., standard errors) were chosen to reflect current peer-reviewed evidence.

Technical Appendix Table 1. Previous studies estimating the relative risk of Guillain-Barré syndrome from influenza vaccination and illness*

Risk, author (reference)	Influenza seasons	Study design	Risk period	Estimated relative risk or odds ratio (95% CI)†
Influenza vaccination				
Lasky et al. (*3*)	1992–1994	Cohort	42 d	1.7 (1.0–2.8)
Juurlink et al. (*4*)	1992–2004	SCCS	2–7 w	1.45 (1.05–1.99)
Hughes et al. (*5*)	1992–2000	SCCS	42 d	0.99 (0.32–3.12)
Tam et al. (*6*)	1991–2001	Case–control	60 d	0.16 (0.02–1.25)
Stowe et al. (*7*)	1990–2005	SCCS	90 d	0.76 (0.41–1.40)
Greene et al. (*8*)	2009–2010	SCRI	42 d	1.3 (0.5–3.8)
Wise et al. (*9*)	2009–2010	Cohort	42 d	1.43 (0.94–1.89)
Kwong et al. (*10*)	1998–2009	SCCS	42 d	1.52 (1.17–1.99)
Galeotti et al. (*11*)	2010–2011	SCCS	42 d	2.1 (1.1–3.9)
Baxter et al. (*12*)	1994–2006	Case-centered	42 d	1.11 (0.39–3.08)
Baxter et al. (*12*)	1994–2006	SCRI	42 d	1.3 (0.75–2.26)
Influenza-like illness				
Tam et al. (*6*)	1991–2001	Case-control	60 d	18.64 (7.49–46.37)
Stowe et al. (*7*)	1990–2005	SCCS	30 d	17.79 (9.77–32.41)
Kwong et al. (*10*)	1993–2009	SCCS	42 d	15.81(10.28–to 24.32)
Galeotti et al. (*11*)	2010–2011	SCCS	42 d	8.7 (4.2–18.3)

*SCCS, self-controlled case series; SCRI, self-controlled risk interval.
†Under the rare disease assumption, Guillain-Barré Syndrome is sufficiently rare that the odds ratio should be a close approximation of the relative risk.

Technical Appendix Table 2. Influenza illness rates from observational studies and control/placebo groups from randomized controlled trials*

Author (reference), study population by age, y	Rate†	Time period (location)	Outcome	Comment
VanTam et al. (*13*)		1966–1984	LCI	Family studies
Children <5	15.3–44.5 (range)			
Children 5–19	16.8–47.7 (range)			
Adults 20–39	16.1–23 (range)			
Adults ≥40	12–21 (range)			
Jefferson et al. (*14*)		1979–2007	LCI	Pooled estimate from all available RCT control arm data
Children <2	9.6 (40/418)			
Children ≤5	19.4 (881/4539)			
Children 6–18	24.9 (175/703)			
Neuzil et al. (*15*)		1974–1999	LCI	RCT control arm data
Children <5	9.5 (95% CI 8.5–10.5)			
Williams et al. (*16*)		2006/2007	LCI	
Health care workers	11.2			
Non–health care workers	10.3			
Kuster et al. (*17*)		1957–2009	LCI	
Health care workers	7.54 (95% CI 4.86–11.70)			
Working adults	5.12 (95% CI 3.08–8.52)			
Jefferson et al. (*18*)		1970–2009	LCI	Pooled estimate from all available RCT control arm data
Healthy adults	2.73 (529/19383)			
Nicholson et al. (*19*) (cited in Jefferson 2010 Cochrane review; analysis 2.2) (*18*)		1992–1994 (UK)	LCI	
Adults ≥60	9.1 (19/209)			
Voordouw et al. (*20*)		1996–1997 (NL)	ICPC codes for influenza	
Adults ≥65	0.35 (32/8911)			
Govaert et al. (*21*)		1991–1992 (NL)	LCI	
Adults ≥60	8.8 (80/911)			
De Villiers et al. (*22*)		2001 (S. Africa)	LCI	
Adults ≥60	7.5 (118/1569)			

*ICPC, International Classification of Primary Care; LCI, laboratory-confirmed influenza; RCT, randomized controlled trial; NL, the Netherlands; S. Africa, South Africa; UK, United Kingdom.
†Rates are expressed as percentages, and when provided by the authors, the 95% CIs or no/total are given in parentheses following the point estimate.

References

1. Gelman A, Carlin JB, Stern HS, Rubin DB. Bayesian data analysis. 2nd ed. Boca Raton (FL): Chapman and Hall; 2004.

2. Briggs A, Claxton K, Sculpher M. Decision Modelling for Health Economic Evaluation. New York: Oxford University Press; 2006.

3. Lasky T, Terracciano GJ, Magder L, Koski CL, Ballesteros M, Nash D, et al. The Guillain-Barré syndrome and the 1992–1993 and 1993–1994 influenza vaccines. N Engl J Med. 1998;339:1797–802. PubMed http://dx.doi.org/10.1056/NEJM199812173392501

4. Juurlink DN, Stukel TA, Kwong J, Kopp A, McGeer A, Upshur RE, et al. Guillain-Barré syndrome after influenza vaccination in adults: a population-based study. Arch Intern Med. 2006;166:2217–21. PubMed http://dx.doi.org/10.1001/archinte.166.20.2217

5. Hughes RA, Charlton J, Latinovic R, Gulliford MC. No association between immunization and Guillain-Barré syndrome in the United Kingdom, 1992 to 2000. Arch Intern Med. 2006;166:1301–4. PubMed http://dx.doi.org/10.1001/archinte.166.12.1301

6. Tam CC, O'Brien SJ, Petersen I, Islam A, Hayward A, Rodrigues LC. Guillain-Barré syndrome and preceding infection with campylobacter, influenza and Epstein-Barr virus in the general practice research database. PLoS ONE. 2007;2:e344. PubMed http://dx.doi.org/10.1371/journal.pone.0000344

7. Stowe J, Andrews N, Wise L, Miller E. Investigation of the temporal association of Guillain-Barre syndrome with influenza vaccine and influenzalike illness using the United Kingdom General Practice Research Database. Am J Epidemiol. 2009;169:382–8. PubMed http://dx.doi.org/10.1093/aje/kwn310

8. Greene SK, Rett M, Weintraub ES, Li L, Yin R, Amato AA, et al. Risk of confirmed Guillain-Barre syndrome following receipt of monovalent inactivated influenza A (H1N1) and seasonal influenza vaccines in the Vaccine Safety Datalink Project, 2009–2010. Am J Epidemiol. 2012;175:1100–9. PubMed http://dx.doi.org/10.1093/aje/kws195

9. Wise ME, Viray M, Sejvar JJ, Lewis P, Baughman AL, Connor W, et al. Guillain-Barre syndrome during the 2009–2010 H1N1 influenza vaccination campaign: population-based surveillance among 45 million Americans. Am J Epidemiol. 2012;175:1110–9. PubMed http://dx.doi.org/10.1093/aje/kws196

10. Kwong JC, Vasa P, Campitelli MA, Hawken S, Wilson K, Rosella LC, et al. Risk of Guillain-Barré syndrome after seasonal influenza vaccination and influenza health-care encounters: a self-controlled study. Lancet Infect Dis. 2013;13:769–76. PubMed http://dx.doi.org/10.1016/S1473-3099(13)70104-X

11. Galeotti F, Massari M, D'alessandro R, Beghi E, Chiò A, Logroscino G, et al. Risk of Guillain-Barré syndrome after 2010–2011 influenza vaccination. Eur J Epidemiol. 2013;28:433–44. PubMed http://dx.doi.org/10.1007/s10654-013-9797-8

12. Baxter R, Bakshi N, Fireman B, Lewis E, Ray P, Vellozzi C, et al. Lack of association of Guillain-Barre syndrome with vaccinations. Clin Infect Dis. 2013;57:197–204. PubMed http://dx.doi.org/10.1093/cid/cit222

13. Van Tam J. Epidemiology of Influenza. In: Nicholson KG WR, Hay AY, editors. Textbook of influenza. Oxford (UK): Blackwell Science; 1998. p 181–206.

14. Jefferson T, Rivetti A, Di Pietrantonj C, Demicheli V, Ferroni E. Vaccines for preventing influenza in healthy children. Cochrane Database Syst Rev. 2012;8:CD004879. PubMed

15. Neuzil KM, Zhu Y, Griffin MR, Edwards KM, Thompson JM, Tollefson SJ, et al. Burden of interpandemic influenza in children younger than 5 years: a 25-year prospective study. J Infect Dis. 2002;185:147–52. PubMed http://dx.doi.org/10.1086/338363

16. Williams CJ, Schweiger B, Diner G, Gerlach F, Haaman F, Krause G, et al. Seasonal influenza risk in hospital healthcare workers is more strongly associated with household than occupational exposures: results from a prospective cohort study in Berlin, Germany, 2006/07. BMC Infect Dis. 2010;10:8. PubMed http://dx.doi.org/10.1186/1471-2334-10-8

17. Kuster SP, Shah PS, Coleman BL, Lam P-P, Tong A, Wormsbecker A, et al. Incidence of influenza in healthy adults and healthcare workers: a systematic review and meta-analysis. PLoS ONE. 2011;6:e26239. PubMed http://dx.doi.org/10.1371/journal.pone.0026239

18. Jefferson T, Di Pietrantonj C, Rivetti A, Bawazeer GA, Al-Ansary LA, Ferroni E. Vaccines for preventing influenza in healthy adults. Cochrane Database Syst Rev. 2010;CD001269. PubMed

19. Nicholson KG, Kent J, Hammersley V. Influenza A among community-dwelling elderly persons in Leicestershire during winter 1993–4; cigarette smoking as a risk factor and the efficacy of influenza vaccination. Epidemiol Infect. 1999;123:103–8. PubMed http://dx.doi.org/10.1017/S095026889900271X

20. Voordouw BCG, van der Linden PD, Simonian S, van der Lei J, Sturkenboom MCJM, Stricker BHC. Influenza vaccination in community-dwelling elderly: impact on mortality and influenza-associated morbidity. Arch Intern Med. 2003;163:1089–94. PubMed http://dx.doi.org/10.1001/archinte.163.9.1089

21. Govaert TM, Thijs CT, Masurel N, Sprenger MJ, Dinant GJ, Knottnerus JA. The efficacy of influenza vaccination in elderly individuals. A randomized double-blind placebo-controlled trial. JAMA. 1994;272:1661–5. PubMed http://dx.doi.org/10.1001/jama.1994.03520210045030

22. De Villiers PJT, Steele AD, Hiemstra LA, Rappaport R, Dunning AJ, Gruber WC, et al. Efficacy and safety of a live attenuated influenza vaccine in adults 60 years of age and older. Vaccine. 2009;28:228–34. PubMed http://dx.doi.org/10.1016/j.vaccine.2009.09.092
